# Angiographic evaluation of coronary artery disease in diabetic patients with and without end‐stage kidney disease

**DOI:** 10.14814/phy2.16180

**Published:** 2024-08-04

**Authors:** Arash Gholoobi, Mahnaz Ahmadi, Saeed Ghoraba, Leila Bigdelu, Mona Najaf Najafi, Vafa Baradaran Rahimi

**Affiliations:** ^1^ Department of Cardiovascular Diseases, Faculty of Medicine Mashhad University of Medical Sciences Mashhad Iran; ^2^ Vascular and Endovascular Surgery Research Center Mashhad University of Medical Sciences Mashhad Iran; ^3^ Clinical Research Development Unit, Imam Reza Hospital Mashhad University of Medical Sciences Mashhad Iran

**Keywords:** coronary angiography, coronary artery stenosis, diabetes, end‐stage kidney disease

## Abstract

The objective of the present investigation was to compare the coronary angiography results in diabetic patients with and without end‐stage kidney disease (ESKD). We included prolonged diabetic patients with ESKD (93 patients) and without ESKD (control group, 126 patients). Angiography of the coronary arteries was performed on all patients. Our results revealed that the ESKD patients tended to have a higher degree of coronary artery stenosis in all parts of LAD (*p* = 0.001, 0.024, and 0.005), proximal and distal RCA (*p* = 0.013, and 0.008), and proximal and distal LCX artery (*p* = 0.001, 0.008) than non‐ESKD patients. Furthermore, we found that the ESKD group had higher significant coronary artery stenosis in the LAD artery (60.5% vs. 39.5%, *p* < 0.001), RCA (60.3% vs. 39.7%, *p* < 0.001), LCX artery (79.5% vs. 20.5%, *p* < 0.001), and LMCA (84.6% vs 15.4%, *p* = 0.002) compared to control group. There was a greater prevalence of multiple vessels coronary artery disease (≥ two) among ESKD patients (29%), compared with the non‐ESKD group (16.8%, *p* < 0.001). Significant coronary artery stenosis was meaningfully higher in asymptomatic diabetic ESKD patients on hemodialysis than non‐ESKD diabetic patients. Coronary angiography may be beneficial in diabetic patients with ESKD regardless of whether they have ischemic symptoms with low complication rate through radial access.

## 1 INTRODUCTION

Diabetes is a widespread chronic disease that causes severe injuries to multiple organs over time, namely the heart, blood vessels, kidneys, eyes, and nerves (Kehlenbrink et al., 2023). Diabetes is considered a critical factor that increases risk of coronary artery disease (CAD), and diabetic patients are more likely to experience cardiovascular events and mortality than non‐diabetic patients (Valensi et al., 2021). In this regard, it has been reported that CAD is responsible for more than 50% of deaths in diabetic patients (Dastani et al., 2022). CAD in diabetic patients remains asymptomatic and undetected until the advanced stages, which leads to extensive coronary atherosclerosis (Ferrannini et al., 2020).

Plenty of evidence emphasizes that cardiovascular disorders are more prevalent in chronic kidney disease (CKD) patients when compared with the general population (Vallianou et al., 2019). Interestingly, the major adverse cardiac events (MACE) as well as all‐cause mortality are conversely correlated by the glomerular filtration rate (GFR) (Miller‐Hodges et al., 2018). In addition, cardiovascular disorders are the most common reason for mortality and morbidity in end‐stage kidney disease (ESKD) patients on hemodialysis (HD) (Zoccali et al., 2023). Patients with ESKD may experience a higher risk of acute coronary syndrome, mortality rate, and have worse prognosis after myocardial infarction (MI) than non‐ESKD patients (Dai et al., 2017). Therefore, detecting CAD early in diabetics and ESKD patients is critical and beneficial in order to alleviate mortality and morbidity in these patients.

Surprisingly, there was a higher incidence of MACE among diabetics with ESKD after acute coronary syndrome than non‐diabetics with ESKD and diabetics without ESKD (Goto et al., 2008). However, the effects of diabetes and ESKD on the extent of CAD are not yet well established. We therefore evaluated and compared coronary angiography results in diabetic patients with and without ESKD in the current study.

## 2 MATERIALS AND METHODS

### 2.1 Ethics

In accordance with university ethics committee policy, this study was approved by Mashhad University of Medical Sciences (approval code. IR.MUMS.fm.REC.1396.274). An informed consent form was provided to all participants and signed by them, and patients were fully informed of the potential benefits and complications.

### 2.2 Study design

The present investigation was a two‐group case–control investigation conducted on all diabetic patients with ESKD (ESKD group) and without ESKD (control group) who underwent coronary angiography in Imam Reza Hospital an affiliate of Mashhad University of Medical Sciences, Mashhad, Iran between February 2018 to May 2019.

### 2.3 Inclusion and exclusion criteria

We recruited prolonged diabetic patients (more than 10 years) with ESKD (GFR <15 mL/min) undergoing HD who were referred for coronary angiography in the “ESKD group.” Additionally, prolonged diabetic patients without ESKD who underwent coronary angiographic evaluation were included in the control group. The indications for angiography in both groups were based on the physician's discretion. The main indication for coronary angiography was reduced LVEF, as a routine practice before renal transplantation in our center and positive imaging studies for ischemia. The stenosis severity was determined eyeball in two orthogonal angiographic views by an expert interventional cardiologist who was blinded to the study patients.

All diabetic patients were known cases for more than 1 year mainly treated with insulin in ESKD group and with oral or injectable anti‐diabetic medications in non‐ESKD group. All patients were receiving high‐intensity statin therapy.

The groups were matched according to age and gender. We excluded patients with collagen‐vascular disease, coagulopathy, and known complications of diabetes such as retinopathy and neuropathy.

Patients with known coagulopathy, who were identified too high risk for bleeding complications, were excluded from the invasive angiographic study. Furthermore, we wanted to have more homogenous group of patients without microvascular complications and focus on whom with macrovascular disease.

### 2.4 Assessment of results

The demographic information of patients, particularly age and sex, and risk factors of cardiovascular disorders such as the previous history of hypertension (HTN), dyslipidemia, and smoking, was documented. Aside from that, left ventricular ejection fraction (LVEF) before angiography, duration of diabetes, duration of HD, serum creatinine (Cr) level, as well as New York Heart Association (NYHA) class were recorded. GFR was calculated using 2021 CKD‐EPI Cr equation (Chen & Shi, 2024).

After that, coronary angiography was performed by an expert interventional cardiologist on all patients. All evaluations were conducted using the Artis zee angiographic unit (Siemenes, AG, Munich, Germany) with less than 50 cc of contrast media (Iodixanol 320, GE healthcare). Angiographic access was obtained mainly by radial route and in some cases through femoral route. Afterwards, the number of involved vessels and extent of right coronary artery (RCA), left anterior descending artery (LAD), left circumflex coronary artery (LCX), obtuse marginal artery (OM), and left main coronary artery (LMCA) stenosis were also recorded. The nonsignificant, moderate, and significant coronary stenosis was considered as <30%, 30%–70%, and >70% coronary diameter stenosis by visual estimation.

### 2.5 Statistics

Statistical analysis was carried out using SPSS version.22. (SPSS Inc., Chicago, Illinois) as well as Graph Pad Prism 8.01 (Graph Pad Software Inc., USA). According to their nature, parametric and nonparametric data were expressed as means ± SD or numbers with percentages. To determine the difference between categorical variables, we used the Chi‐square test. Furthermore, student's *t*‐tests were utilized for parametric data and Mann–Whitney *U* tests for nonparametric data when appropriate to compare continuous variables. Statistical significance was identifying by *p* values (*p*) ≤ 0.05.

## 3 RESULTS

### 3.1 Clinical characteristics

The study included 219 patients, 93 of whom were patients with ESKD and 126 of whom were in the control group. Additionally, the total mean age of participants was 63.38 ± 12.85 years. There was a good distribution of demographic characteristics in both studied groups, including age, sex, smoking, history of HTN and dyslipidemia, as well as duration of diabetes (Table 1). The cause of ESKD in our patients were mainly HTN, diabetes, or both of these disorders. However, as compared to the ESKD group, the control group had a significantly greater LVEF (*p* = 0.003). Additionally, none of our patients had proteinuria.

**TABLE 1:** The baseline characteristics and clinical findings.

Characteristics		ESKD group (*N* = 93)	Control group (*N* = 126)	*p*‐value
Age (Mean ± SD), years		54.98 ± 12.20	57.03 ± 12.40	0.224^a^
Sex (*n*, %)	Male	47 (50.5)	60 (47.6)	0.386^b^
	Female	46 (49.5)	66 (52.4)	
Hypertension (*n*, %)		22 (23.7)	28 (22.2)	0.464^b^
Smoking (*n*, %)		14 (15.1)	11 (8.7)	0.108^b^
Dyslipidemia (*n*, %)		31 (33.3)	34 (27)	0.193^b^
Duration of diabetes (Mean ± SD), years		16.47 ± 5.38	15.58 ± 6.75	0.353^a^
Ejection fraction (Mean ± SD), %		51.29 ± 7.79	54.4 ± 6.97	0.003^a^
Creatinine (Mean ± SD), mg/dl		–	1.12 ± 0.3	–
GFR (Mean ± SD), ml/min/1.73 m^2^		–	71.2 ± 22.3	–
GFR Grade	1	–	32 (25.4%)	–
	2	–	51 (40.5%)	
	3a	–	25 (19.8%)	
	3b	–	17 (13.5%)	
	4	–	1 (0.79%)	
	5	93 (100%)	–	
NYHA (n, %)	Class I	67 (38.3)	108 (61.7)	0.01^b^
	Class II	26 (59.1)	18 (40.9)	
Duration of dialysis (Mean ± SD), years		4.84 ± 2.64	–	–

Abbreviations: ESKD, End‐stage kidney disease, NYHA, New York Heart Association functional classification.

^a^ Comparing between the ESKD and control groups using Student's *t*‐test.

^b^ Comparing the ESKD and control groups using Chi‐square test.

### 3.2 The degree of stenosis in LAD, RCA, LCX, OM, and LMCA in ESKD and control groups

**FIGURE 1:**
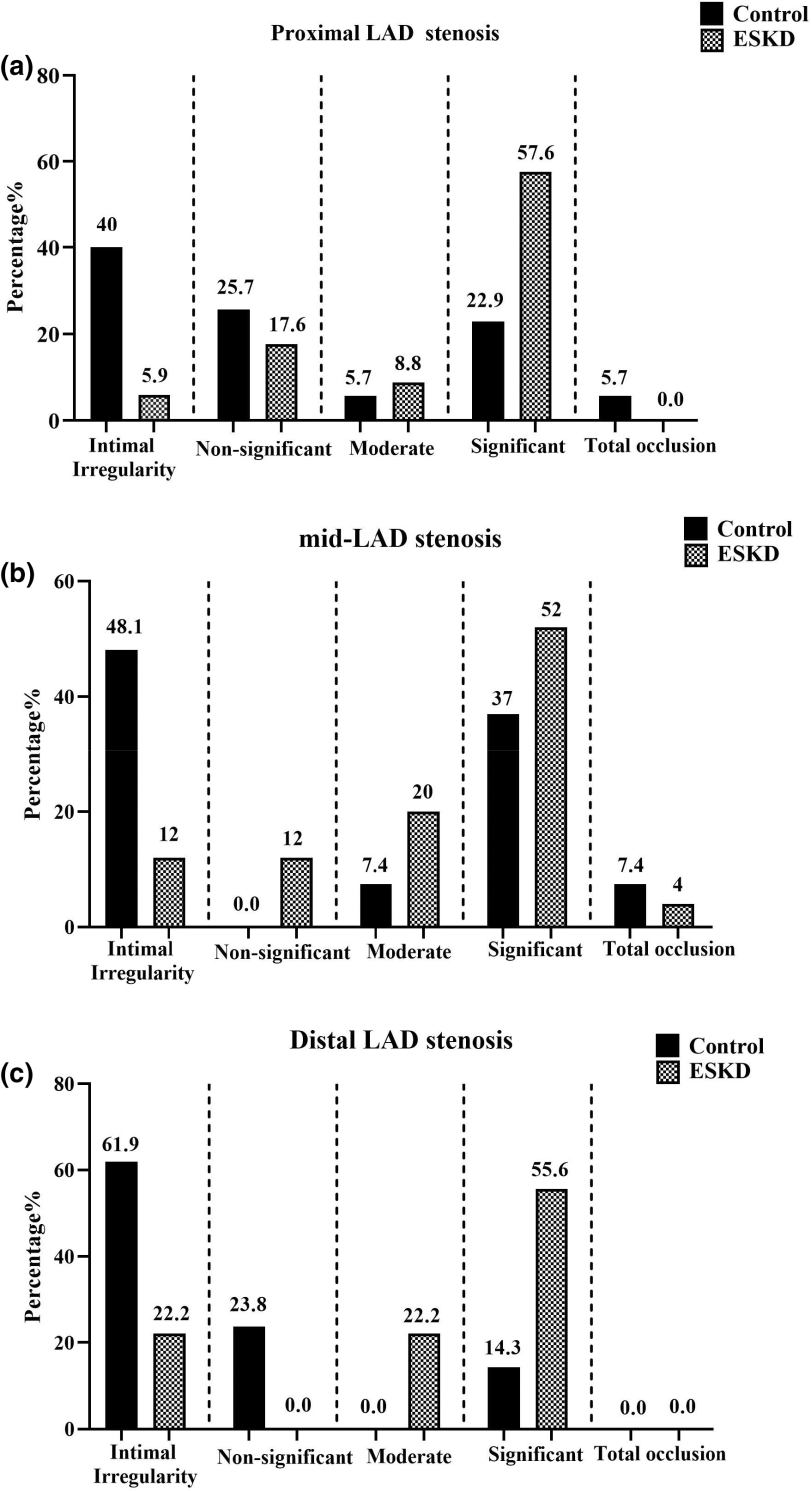
The degree of stenosis in the (a) proximal, (b) mid‐part, and (c) distal LAD in ESKD and control groups. Data were expressed as a percentage of patients; ESKD, End‐stage renal disease; LAD, Left anterior descending artery.

As compared to the control group, the degree of stenosis in proximal (*p* = 0.001, Figure 1a), mid‐part (*p* = 0.024, Figure 1b), and distal part of LAD was significantly higher in the ESKD patients (*p* = 0.005, Figure 1c).

**FIGURE 2:**
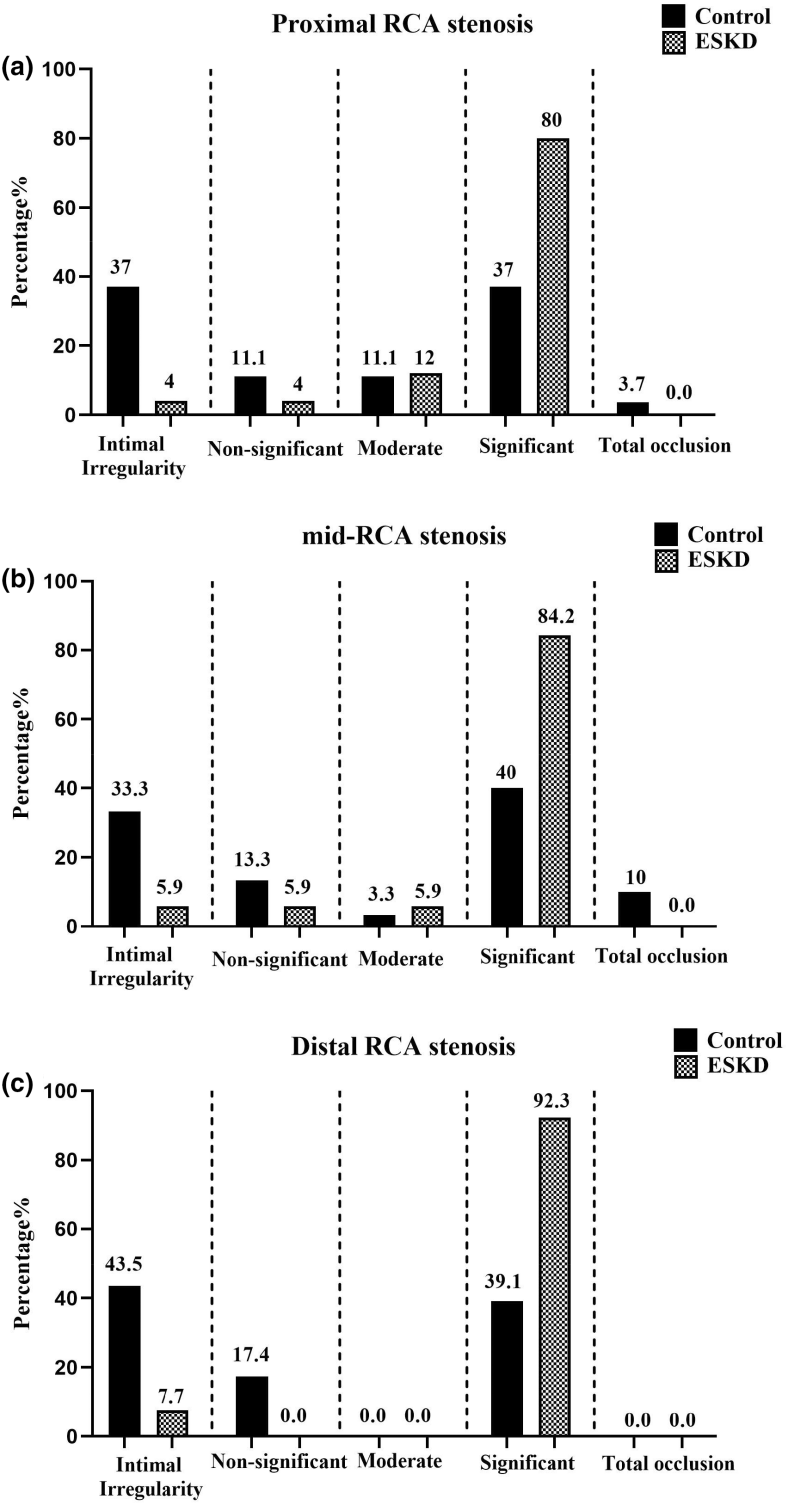
The degree of stenosis in the (a) proximal, (b) mid‐part, and (c) distal RCA in ESKD and control groups. Data were expressed as a percentage of patients; ESKD, End‐stage renal disease; RCA, Right coronary artery.

The degree of stenosis in the proximal, mid part, and distal RCA is shown in Figure 2. In more detail, a higher degree of stenosis was observed in the ESKD patients in proximal (*p* = 0.013, Figure 2a) and distal (*p* = 0.008, Figure 2c) than the control group. However, no remarkable difference was reported in the mid part RCA among the two studied groups (*p* = 0.051, Figure 2b).

**FIGURE 3:**
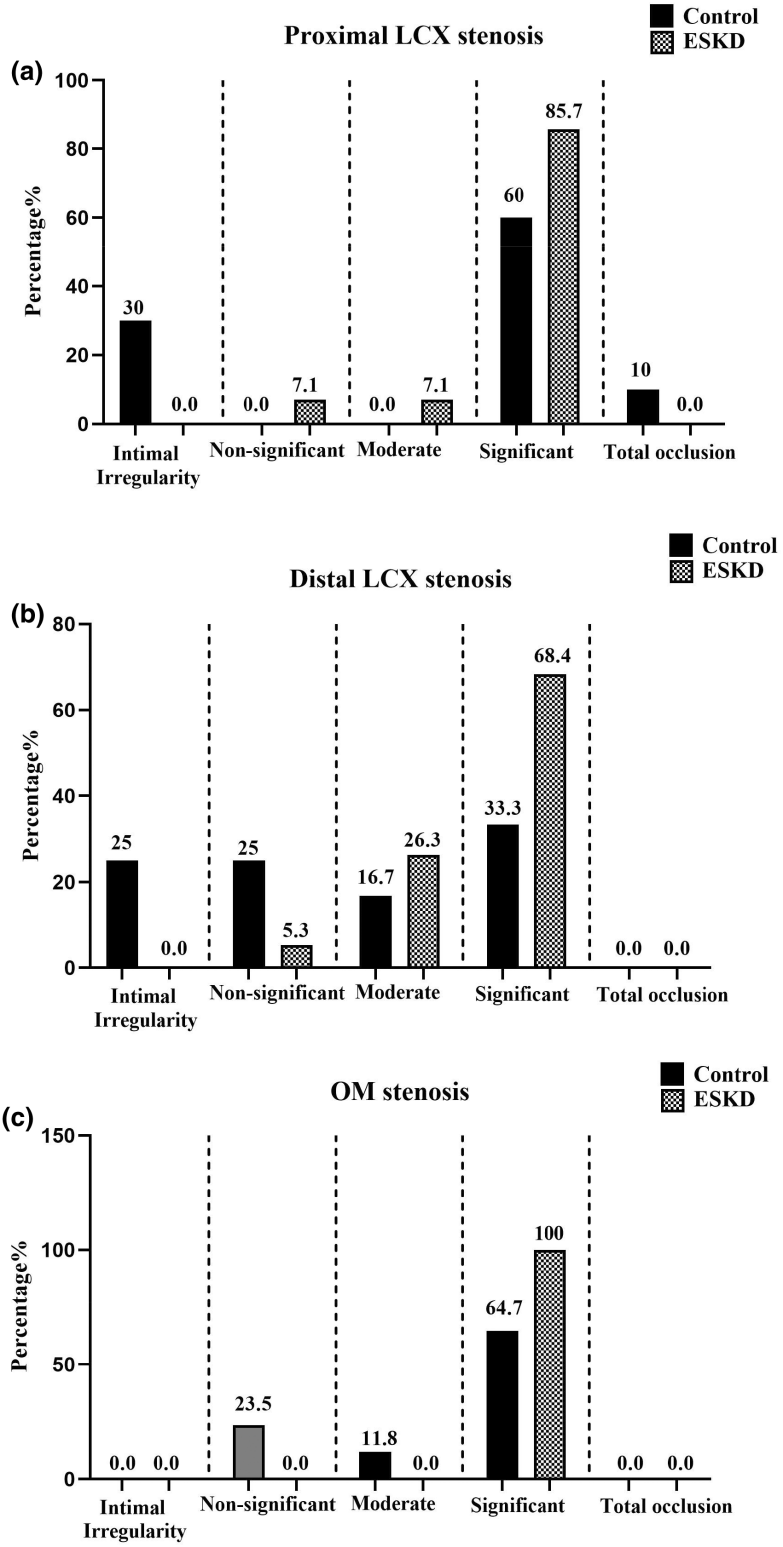
The degree of stenosis in the (a) proximal LCX, (b) distal LCX, and (c) OM in ESKD and control groups. Data were expressed as a percentage of patients; ESKD, End‐stage renal disease; LCX, Left circumflex coronary artery; OM, Obtuse marginal artery.

As represented in Figure 3, the degree of stenosis was meaningfully higher in proximal (*p* = 0.001, Figure 3a) and distal (*p* = 0.008, Figure 3b) LCX in the ESKD group when compared to the control group. Additionally, a greater degree of OM stenosis was observed in the ESKD group than in the control group (*p* = 0.028, Figure 3c).

Aside from that, Table 2 shows the significant coronary stenosis in the two studies groups. We found that the significant coronary stenosis in the LAD (60.5% and 39.5%, *p* < 0.001), RCA (60.3% and 39.7%, *p* < 0.001), LCX (79.5% and 20.5%, *p* < 0.001), and LMCA (84.6% vs. 15.4%, *p* = 0.002) was strikingly higher in the ESKD patients than those of the control group.

**TABLE 2:** The significant coronary stenosis in the ESKD and control groups.

Coronary vessel	ESKD group (*N* = 93)	Control group (*N* = 126)	*p*‐value^a^
LAD (%)	60.5	39.5	<0.001
RCA (%)	60.3	39.7	<0.001
LCX (%)	79.5	20.5	<0.001
LMCA (%)	84.6	15.4	0.002

Abbreviations: ESKD, End‐stage kidney disease, LAD, left anterior descending artery, LCX, Left circumflex coronary artery, LCMA, Left main coronary artery; RCA, Right coronary artery.

^a^ Comparing the ESKD and control groups using Chi‐square test.

### 3.3 The distribution of CAD and final diagnosis in the studies groups

**FIGURE 4:**
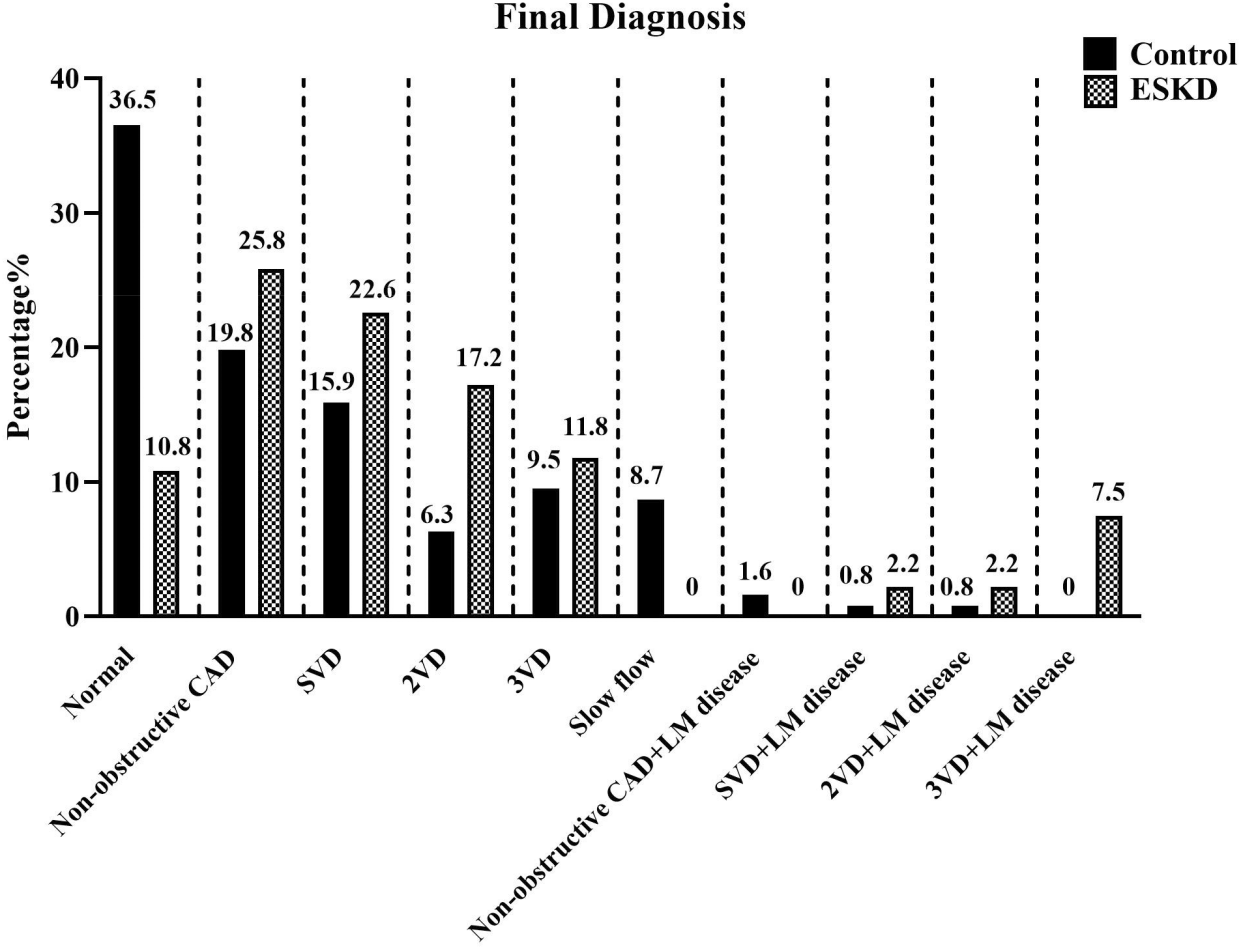
The final diagnosis in the ESKD and control groups. Data were expressed as a percentage of patients. CAD, Coronary artery disease; ESKD, End‐stage renal disease; LM, Left‐main; SVD, Single vessel disease, 2VD, Two‐vessel disease, 3VD, Three‐vessel disease.

The coronary anatomy and final diagnosis in the two investigated groups are presented in Figure 4. Based on our findings, the extent of coronary involvement in the ESKD patients was more remarkable than those of the control group (*p* < 0.001). The patients with nonobstructive CAD were 19.8% in control and 25.8% in the ESKD group. Furthermore, patients with single‐vessel disease, two‐vessel disease, and three‐vessel disease were 15.9%, 6.3%, and 9.5% in the control group and 22.6%, 17.2%, and 11.8% in the ESKD group.

Fortunately, no complications occurred in any of the patients and uneventfully discharged the same day or the next morning after the procedure.

## 4 DISCUSSION

To the best of our knowledge, the current investigation was the first to examine and compare the severity of CAD in prolonged diabetic patients with and without ESKD. Our results revealed that the ESKD patients tended to have a higher degree of coronary stenosis than the control group in all parts of LAD, proximal and distal RCA, and proximal and distal LCX. Furthermore, we found that the significant coronary stenosis in the LAD, RCA, LCX, and LMCA in the ESKD patients was strikingly greater in comparison with the control group. Moreover, the multi‐vessel CAD (≥ two) was increasingly prevalent in ESKD patients comparing to those in the control group.

Several pieces of evidence emphasized that ESKD stimulates the risk of cardiovascular disease‐related mortality and morbidities. Surprisingly, ESKD patients had increased levels of inflammatory and pro‐thrombotic factors, including C‐reactive protein (CRP), lipoprotein (a), interleukin‐6 (IL‐6), and factor VIII (Gelev et al., 2008; Rahmanian‐Devin et al., 2021). In addition, higher CRP levels were related to higher arterial intima calcification and arterial media calcification in long‐term HD patients with ESKD (Marinelli et al., 2011).

Obstructive coronary disease is diagnosed using coronary angiography as the gold standard (Jiangping et al., 2013). Although it is an invasive procedure and may lead to some complications, invasive coronary angiography is recommended in ESKD patients rather than other detecting methods (De Lima et al., 2003; Enkiri et al., 2010). Furthermore; in expert hands it can be done with a low contrast media volume and complication rate. In this regard, Jayme and coworkers supported that coronary angiography can better detect CAD and predict the cardiac events in patients who are candidate for kidney transplant than noninvasive methods such as myocardial scintigraphy as well as dobutamine stress echocardiography (De Lima et al., 2003). Similarly, Gang et al. supported that CAD can be accurately predicted with coronary angiography in patients with diabetes type 2 candidate for kidney transplant (Gang et al., 2007).

Coronary CT angiography (CCTA) is another noninvasive technique that is also recommended for examining CAD. However, coronary artery calcification with coronary calcification scores higher than 400 HU is highly prevalent in ESKD patients (Cano‐Megías et al., 2019). It has been suggested that coronary calcification may prevent the precise interpretation of the CCTA (Jablonski & Chonchol, 2013). In addition, CCTA utilize the higher contrast volume than coronary angiography which may also limit the use of CCTA in ESKD patients (Oda et al., 2019). Therefore, CCTA is not an appropriate method in evaluating majority of diabetic ESKD patients and invasive coronary angiography is recommended in these patients (Kassab & Doukky, 2022).

Based on the findings of this study, the significant coronary stenosis in the LAD, RCA, LCX, and LMCA of the ESKD group was meaningfully greater as compared with the control group. Interestingly, the prevalence of multi‐vessel CAD (≥ two) was also greater in ESKD patients (29%) comparatively to the control group (16.8%). In accordance with our findings, Ohtake and colleagues figured out the coronary angiography results of 30 patients with stage five CKD who are asymptomatic and don't have a history of angina or MI. They noticed that 53.3% of patients had significant coronary artery stenosis, with five patients suffering from severe coronary artery stenosis (≥90%). Moreover, 62.5% of asymptomatic stage five CKD patients had single‐vessel disease, 25% had two‐vessel, and 12.5% had three‐vessel disease. They suggested that coronary angiography in stage 5 CKD patients may be beneficial without regard to having ischemic symptoms (Ohtake et al., 2005). Similarly, an analysis of 368 asymptomatic patients with ESKD on HD found that 45% had CAD. In fact, there were 17% with three‐vessel disease, 11% with two‐vessel disease, and 17% with a single‐vessel disease. Additionally, they showed that 5.2% had significant LMCA, 33.7% had considerable LAD stenosis, 28% had significant LCX stenosis, and 26.9% had significant RCA stenosis. They supported that significant coronary artery stenosis prevalence is high among asymptomatic ESKD patients. The survival rate was lowest among patients with two‐ or three‐vessel disease in the next 5 and 10 years (Mohamed et al., 2020).

Hayashi et al. investigated 60 asymptomatic CKD patients about to start renal replacement therapy with no previous history of angina or MI. They figured out that 43.8% of patients suffered from significant coronary artery stenosis, and the prevalence of single‐, two‐, and three‐vessel disease was 22.9%, 28.6%, and 48.5%, respectively (Hayashi et al., 2008). On the other hand, Lim and coworkers determined the results of 82 ESKD patients on dialysis who underwent coronary angiography due to MI. They demonstrated that 39% had three‐vessel disease, 28.1% had two‐vessel disease, and 19.5% had single‐vessel disease (Lim & Lee, 2020). These results are consistent with our findings regarding the increased prevalence of coronary artery stenosis in asymptomatic ESKD patients.

Previous studies suggested that even mild CKD is considered a coronary risk factor (Khameneh Bagheri et al., 2022; Moravvej et al., 2022). In this regard, diabetes and CKD are predisposing factors that contribute to the development of vascular calcification, a condition associated with calcium and phosphorus deposition (Giachelli, 2004). Atherosclerotic obstructive plaque in diabetes and CKD is associated with increased coronary calcification along with media thickening. Patients with diabetes and CKD without dialysis showed a higher prevalence of vascular calcifications, similar to ESKD patients (Janda et al., 2015). Consistently, Beddhu and coworkers supported that the risk of MI and death was propagated in the case of moderate renal failure. Patients with the lowest GRF quartile had 1.5 fold elevated MI, 4.2 fold increased death, as well as 2.8 fold stimulated death/MI than the highest GFR quartile (Beddhu et al., 2002).

Similarly, Laskey and coworkers evaluated a comparison of coronary angiography in patients with diabetes mellitus as well as those without. Diabetes patients had an increased number of clinical and angiographic risk factors, in‐hospital and one‐year mortality rates, and a need for repeat revascularization compared to nondiabetic patients (Laskey et al., 2002). Scognamiglio and coworkers determined the coronary angiographic results of 1899 type 2 diabetes patients who are asymptomatic. They categorized them as having ≥ two associated CAD risk factors (group A, 62%) or having ≤1 associated CAD risk factor (group B, 38%). According to their findings, both groups had a similar prevalence of significant CAD (64.6% and 65.5%, *p* = 0.81). However, One‐, two‐, and three‐vessel diseases were represented in remarkably different proportions in group A (70.6%, 21.8%, and 7.6%, respectively) than group B (46.3%, 20.4%, and 33.3%, respectively, *p* < 0.001) (Scognamiglio et al., 2006). These studies highlighted the importance of renal failure and diabetes and may confirm our results regarding the higher extent of CAD in patients suffering from diabetes with ESKD than without ESKD.

## 5 CONCLUSION

In summary, the significant coronary stenosis in the LAD, RCA, LCX, and LMCA was remarkably greater in the asymptomatic and diabetic ESKD patients on HD than in diabetic non‐ESKD patients. Moreover, multi‐vessel CAD (≥ two) was more prevalent in ESKD patients comparatively to the control group. Therefore, coronary angiography may be beneficial in diabetic patients with ESKD regardless of whether they have ischemic symptoms.

## AUTHOR CONTRIBUTIONS

**Arash Gholoobi**: Conceptualization, Methodology, Funding acquisition, Supervision, Writing—review and editing; **Mahnaz Ahmadi**: Investigation, Data Curation, Resources; **Saeed Ghoraba**: Investigation, Data Curation; Leila Bigdelu: Supervision, Investigation, **Mona Najaf Najafi**: Formal analysis, Software; **Vafa Baradaran Rahimi**: Formal analysis, Writing—original draft, Writing—review and editing.

## FUNDING INFORMATION

This study was financially supported by the research council of Mashhad University of Medical Sciences (Grant Number: 960123).

## CONFLICT OF INTEREST STATEMENT

Conflicts of interest were not present.

## ETHICS STATEMENT

In accordance with university ethics committee policy, this study was approved by Mashhad University of Medical Sciences (approval code. IR.MUMS.fm.REC.1396.274). An informed consent form was provided to all participants and signed by them, and patients were fully informed of the potential benefits and complications.

## Supporting information


Data S1.

## Data Availability

The data sets used for the current study are available from the corresponding author upon reasonable request.
