# Supplementary material for: Angiographic evaluation of coronary artery disease in diabetic patients with and without end‐stage kidney disease
Source: Physiol Rep. 2024 Aug 4;12(15):e16180. doi: 10.14814/phy2.16180 (PMC11298246; doi:10.14814/phy2.16180)
Supplement: Supplementary file 1 — Data S1. [file PHY2-12-e16180-s001.docx]

# 27 June 2024

**Editor in Chief
Physiological Reports**

**Josephine C. Adams, University of Bristol, UK**

**(Cell and Molecular Physiology, Cancer)**

re: **Angiographic evaluation of coronary artery disease in diabetic patients with and without End-stage renal disease**

Dear **Editor**

I will be delighted if you reconsider the above manuscript for publication in "**Physiological Reports**". In our best, we addressed all the issues and highlighted by yellow colour. The work is an original clinical study and has not been published or submitted for publication at the present time. However, our team believes that it gains sufficient citations for your valuable journals and for us after the publication of the manuscript.

I look forward to hearing from you.

Yours sincerely

**Vafa Baradaran Rahimi**, Pharm D, PhD;

Assistant Professor; Department of Cardiovascular Diseases, Faculty of Medicine, Mashhad University of Medical Sciences, Azadi Sq., Vakil Abad Highway, Mashhad, 9177948564, Iran; [baradaranrv@mums.ac.ir](mailto:baradaranrv@mums.ac.ir), [vafa_br@yahoo.com](mailto:vafa_br@yahoo.com)

ASSOCIATE EDITOR'S COMMENTS TO THE AUTHORS (IF ANY):

The authors have made some revision to their manuscript but they have failed to address the Associate Editor Comments provided with the reviewer comments.
I provide the comments below that must be addressed in a subsequent revision:
Further comments from the Editorial Board
- please define ethics approval for this study and how patients were consented

Response: Thank you for your scientific comments. It was added accordingly.

**Ethics**

In accordance with university ethics committee policy, this study was approved by Mashhad University of Medical Sciences (approval code. IR.MUMS.fm.REC.1396.274). An informed consent form was provided to all participants and signed by them, and patients were fully informed of the potential benefits and complications.

- please outline the cause of end-stage kidney disease in the patient population.

Response: Thank you for your scientific comments. The cause of ESKD in our patients were mainly hypertension, diabetes, or both of these disorders. It was added accordingly.

The cause of ESKD in our patients were mainly hypertension, diabetes, or both of these disorders.

- note that current nomenclature is ESKD not ESRD

Response: Thank you for your scientific comments. It was corrected accordingly.

- define proteinuria in the "control" group with diabetes as this feature alone (even in the context of normal serum creatinine) is associated with increased risk of CV disease.

Response: Thank you for your scientific comments. None of our patients had proteinuria. It was added accordingly.

I also note that creatinine is not necessarily within the normal range for the control group, therefore please define stages of CKD in this cohort.
Response: Thank you for your scientific comments. The GFR was calculated stages of CKD were presented at Table 1.

Reviewer #2 also has concerns regarding data analysis and the authors must address this in the revised manuscript.
There are a number of sentences that are rather casual rather than scientifically written eg "Inclusion and exclusion criteria "...known cases for a long time...". Please be more accurate in these descriptors.
Response: Thank you for your scientific comments. It was corrected accordingly.

REVIEWER(S)' COMMENTS TO THE AUTHORS:

Reviewer #2 (Comments to the Author (Required)):

Thank you for your responses. I remain concerned regarding the rigor/reliability of the data. Quantifying the sole outcome variable i.e. stenosis "by eyeball" and "estimation" by a single investigator lacks precision raises concern of reproducability.

Response: Thank you for your scientific comments. However, the interventional cardiologist had more than 15 years’ experience in catheterization laboratory. According to your concern, we randomly selected some cases and reviewed by another interventional cardiologist and the results were the same.
